# That Which They Will Not See: Climate Denial as a Vector of Epistemological Crisis in the Contemporary United States

**DOI:** 10.1080/00141844.2023.2242599

**Published:** 2023-08-08

**Authors:** Susannah Crockford

**Affiliations:** University of Exeter, UK

**Keywords:** Climate change, anthropology, agnotology, epistemology, ethnography

## Abstract

Climate denial continues as a cultural epistemology for anthropogenic climate change in the United States, despite worsening impacts. This article offers an ethnographic account of rural areas in three states in the southern US – Arizona, Louisiana, and Missouri – based on long-term participant observation and interview data. Engaging with the literature on agnotology, the social construction of ignorance, the argument is made that this literature as it pertains to climate denial does not go far enough in accounting for the persistence of the rejection of climate science. Theoretically drawing from anthropological work on the incommensurability of paradigms, the argument is based on a tripartite construction of denial as produced through an interaction of a cultural norm of radical empiricism, a political-media ecosystem funded by fossil fuel companies, and a cosmological schema derived from conservative white evangelicalism. The result of this process is an epistemological crisis in contemporary American society.

## Introduction

Anthropogenic climate change is rapidly altering Earth’s ecosystems, undermining the survival of the living organisms currently adapted to those ecosystems. Climate scientists hold a consensus view on this process, and yet in 2018 when research for this article began, around half of respondents to polls and surveys in the US continued to state that they did not believe it (Hall *et al*. 2018: 55). Analysis often frames this as a problem of denial. Climate change is not ‘believed in’, stymying effective ameliorative action. Agnotology frames climate denial as a form of ignorance that has been purposefully produced for specific reasons (Proctor 2008: 16). Sociological literature on climate denial describes a top-down construction, in which vested interests, particularly corporations that profit from the extraction, trade, and use of fossil fuels, continue to both benefit from and actively produce the ignorance of the masses (Oreskes & Conway 2010; Norgaard 2011; Washington & Cook 2011). Despite intentions to move beyond a positivist framing of ignorance, focus dwells on an information deficit; the social production of ignorance through the manipulation of evidence by bad actors blocks the truth; or emotional blockages render people incapable of accepting the truth. Furthermore, it is unclear whether knowledge of climate science leads in any linear way to action to mitigate climate change. In her foundational study of climate denial in Norway, Kari Norgaard (2011: xvi–xvii) describes how her interlocutors were well aware of and in accordance with scientific understandings of anthropogenic climate change, and yet they continued to act as if they were not. An important question for the anthropology of knowledge can therefore be posed; how is climate knowledge constituted, and how does it connect to everyday action?

Drawing on long-term ethnographic engagement in the US South and situated theoretically in the anthropology of knowledge and of climate, I ask what cultural assumptions underlie knowledge construction processes of those who are labelled as ‘in denial’. For Kath Weston (2017: 107) and Robin Globus Veldman (2019: 1), climate denial is a loaded term, even classist, and they prefer the more neutral seeming ‘scepticism’. Yet this preference sits awkwardly alongside the preference of libertarian think tanks that profit from its promulgation, who use the scientific prerogative of being sceptical to affirm their approach as more scientific than that of 97% of climate scientists (Barla & Bjork-James 2021: 380). In this article, I reframe climate denial as a vector of an ongoing epistemological crisis. Moving beyond agnotology, I examine how cultural epistemologies are constituted through drawing together anthropological theory on knowledge and climate (Tambiah 1990; Mitchell 2002; Norgaard 2011; Dumit 2012; Barnes *et al*. 2013; Lahsen 2015; Fiske 2016; Weston 2017; Liboiron 2021; Dewan 2022; Vaughn 2022).

Francis Beer and Robert Hariman (2020: 20) argue that the Covid-19 pandemic exposed an epistemological crisis of stark knowledge disparities between vernacular and scientific explanations of causality and solutions. Catastrophes, such as the pandemic, lay bare social fault lines – ‘problems that already were known but partially denied’ (23) – while also undermining modes of understanding, such as description and prediction, that might articulate a coherent response. Competing scientific and vernacular explanations of existential problems lose meaning in a disrupted context, in which dissonance is interpreted as manifesting harm. For my argument, it is relevant that rejection or questioning of the aetiology of Covid-19 or the efficacy of vaccines also became framed as a problem of ‘denial’ from 2020 onwards.^1^ I extend Beer and Hariman’s definition of epistemological crisis to include climate change, another ongoing catastrophe that reveals pre-existing problems while attenuating modes of understanding. Denial is a vector of this crisis in the biological sense, transmitting a social pathogen.

I situate this reframing in conversation with anthropological work on white evangelical Protestants, a group associated in particular with climate denial, either through opposition to secular culture or end-times chronotope (Veldman 2019; Bjork-James 2023; Ricker 2020). Those named climate deniers do not simply dwell in a socially constructed ignorance but trust in different forms of moral authority, often religiously informed, and give credit to different forms of expertise. While a rationalised placeless expertise aimed at perfecting societies has become the norm in the name of modernising and developing, local peoples have refracted this ‘locationless logic’ (Mitchell 2002: 15). Similarly, expressing doubt over scientific connections between global temperatures and increased carbon emissions can be read as ‘a different epistemology of nature and culture’ that fits into a broader American way of knowing and living with climate (Fiske 2016: 319).

It is this different epistemology of nature and culture that I elucidate in this article, with the intent of parsing conceptual difference rather than othering. In the places and spaces in the US South in which I asked about climate change, the answers alluded to different epistemological systems, supported by contextualised formulations of rationality and epistemic authorities. Natural and supernatural causes of climate change predominated over the human causation authorised in climate science (Hulme 2016: 41–46). In part, this article asks which explanations have authority and why. Epistemological systems constitute a complex structured network of what is known, what can be known, and what is not thought about.

The cultural epistemology underlying climate denial has three interlinked components, in my argument. For the first, I introduce the term *radical empiricism* to refer to a dissociation of knowledge produced through scientific methods from immediate human experience, distinct from pragmatist philosopher William James’ (1912) use of the term. Unlike Weston’s (2017: 114) use of embodied empiricism, radical empiricism shifts focus from the sensory body to a cultural disjuncture between knowledge produced by scientific institutions and an embedded awareness of lived climates. Radical empiricism is produced through interaction with the second component: a *political-media ecosystem* of counternarratives eroding trust and authority. An interconnected system beyond any specific news channel or think tank it incorporates, this ecosystem is self-referential, with its own experts, its own sources, its own trusted spokespeople. Moreover, it has been purposefully mobilised to create a sense of in-group belonging and oppositional stance toward those outside of this ecosystem. The political-media ecosystem leverages the third component: an underlying *cosmological schema* that envisions a naturalised hierarchy ordering the appropriate place of God, humans, and nature.

I argue that this cultural epistemology impedes action on climate change in the US South. Anthropologists have long argued that knowledge does not predicate specific courses of action (e.g. Evans-Pritchard 1937; Needham 1972). Concepts and definitions are fluid and multivalent, and connections between knowledge and action often lack coherence. In terms of knowledge of climate change, how this term is constituted differs among climate scientists and lay publics. Climate scientists often use a restrictive definition hinging on the association between increasing atmospheric CO_2_ and other greenhouse gases and increasing average global temperature. For clarity, in this article I refer to this restrictive definition as anthropogenic climate change. However, it is important to note that climate scientists are not monolithic, and variation exists between scientists on how to study climate change and how significant its various impacts are (Lahsen 2015). Reifying a distinction between weather and climate, some physical scientists insist that anthropogenic climate change is invisible, and what people perceive is only its impacts or manifestations (Rudiak-Gould 2013: 121).

The weather-climate distinction and the restrictive definition of anthropogenic climate change as knowable only by climate scientists through numerical measurements and computational modelling has been questioned by anthropologists (Barnes *et al.* 2013; Weston 2017; Dewan 2022). In the US South, the concept of ‘climate change’ often implies a political position, yet vernacular climate knowledge still incorporates an array of climatic, environmental, and meteorological changes. The term ‘climate’ mediates specialist and vernacular definitions, drawing together sensory experiences of weather with cultural ways of living with weather (Hulme 2016: 6–8). Mike Hulme therefore argues that the term ‘climate’ operates to stabilise and normalise human experience of meteorological and atmospheric processes.

How different people constitute climate knowledge varies, and yet some are framed as in denial, and others are not. The vast chasm that separates people in the Global South and North and their respective contributions to atmospheric carbon levels belies any meaningful comparison between their climate knowledges. The United States is responsible for 20% of global emissions of carbon since 1850, in an analysis of cumulative emissions from fossil fuels, forestry, and land use.^2^ As argued by Thomas Friedrich (2020) regarding Palawan in the Philippines, a lack of information does not hinder climate-friendly behaviour. Those who live in the Global North, especially in the United States, benefit from a surfeit of information about climate change, while nevertheless continuing to contribute ever more to the problem. This points toward the core of climate justice; the corollaries and culpability of climate change are inequitably distributed along axes of colonialist extraction and consumption (Sultana 2022).

The silences of those living in the Global North on climate change mirror those other omissions produced through profiting from racial capitalism (Mills 1997: 20). Race operates as a condition of vulnerability to both ongoing impacts and proposed solutions to anthropogenic climate change (Vaughn 2022). Systemic white supremacy, referring to whiteness as a dominant category in a racialised hierarchical structure of capitalism (not the behaviour of individual white supremacists), coproduced with colonialism the socioeconomic conditions of domination underlying anthropogenic climate change. Racial capitalism generated an ideology of freedom for white people, and most of all for white, wealthy men. But for white people to enjoy this freedom, they have to deny its nature because ‘very few people willingly embrace what they perceive to be evil’ (Stovall 2021: 7). The prosperity that bought this freedom sustains a sense of innocence that absolves hegemonic whiteness and masculinity. Anthropogenic climate change entails an existential threat for which systemic white supremacy has no epistemological space. Those who benefit the most from racial capitalism have the most to gain from refusing to acknowledge this threat.

Rather than reiterating the elephant-in-the-room metaphor (Zerubavel 2006: 14), I address denial in terms of what Thomas Kuhn (1962: 150) theorised as the incommensurability of paradigms. Different paradigms have modes of understanding and ways of communicating specific to those paradigms; when different paradigms come into contact, they tend to talk past each other because they are incommensurable. Instead, contact between paradigms produces a crisis that leads to paradigm shift. One paradigm prevails over others and then what Kuhn calls ‘normal science’ takes place, posing and solving problems within the established paradigm. Bringing awareness to the instability of knowledge, where boundaries of knowledge shift, illuminates differing epistemological paradigms (Dumit 2012: 36). Climate science is a different paradigm, with different forms of evidence, explanatory models, and experimental procedures, yet operating in the same social reality as the cultural epistemology labelled denial. Different paradigms in the same social reality can be incommensurable (Tambiah 1990: 124–126). Comparing ‘scientists and anticolonialists, Elders and peer reviewers’, Indigenous scientist Max Liboiron names their worlds incommensurable, with separate evaluatory frameworks (2021: 33). What can be said, and understood, in one context, cannot be said in another context, or at least, it is not understood in the same way. When interacting in the same social reality, the friction between those incommensurable paradigms produces an epistemological crisis, an ongoing shift in unstable knowledge boundaries.

In this article, I trace a cultural epistemology of denial using ethnographic material from Arizona, Missouri, and Louisiana introduced through a series of automotive arrival stories to contextualise each location.^3^ Through long-term participant observation in the US South since 2012, I encountered primarily a discursive absence surrounding anthropogenic climate change. In a targeted series of semi-structured interviews in 2018 in Arizona, Missouri, and Louisiana, I asked respondents directly about climate change and found a marked reluctance to talk about it, expressing fears that I was party to a Democratic plot or ‘some blog’ intending to ridicule them. My social position as a cultural and political outsider, since I am British, but still close enough to normative racial and linguistic categories of conservatives in the US South, since I am white and speak English, helped ameliorate these fears and enable access to my field-sites. As did travelling with my mother, herself an Anglican minister, and my young son, which situated me in a Christian kin group and made me less suspicious to rural conservatives than a woman travelling alone might have been.

Projections indicate that climate impacts will affect these US states most severely. Arid Arizona is afflicted by excessive heat, riparian Missouri by flooding, and coastal Louisiana by more intense hurricanes (Loomis 2017; Farzhan 2021; Schleifstein 2022). These exposures are exacerbated by a lack of infrastructure and mitigation investment due to state and local governments that are friendly to business and hostile to environmental regulation. Arizona, Missouri, and Louisiana are exposed to extreme weather events exacerbated by anthropogenic climate change, and further vulnerabilised by their local political complexions. In these states, anthropogenic climate change is both severe in its effects and denied or minimised politically and socially, bringing the dynamics constituting the ongoing epistemological crisis into sharp relief.

## I’ll Believe It When I See the Water Rise on the Dock: Erosion and Experience in South-East Louisiana

**Figure 1.**
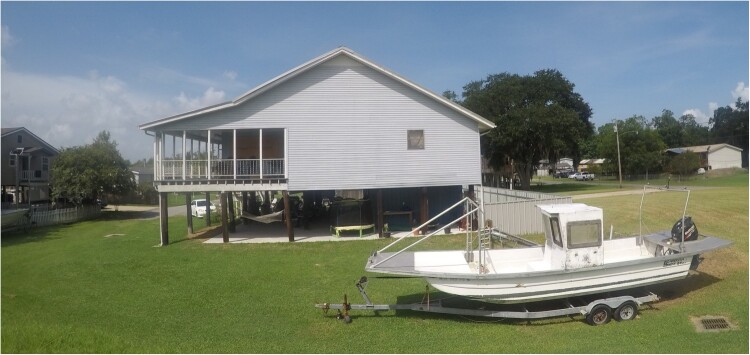
Raised houses in Jean Lafitte, Louisiana. Author copyright.

Driving south from New Orleans towards the Gulf Coast, as you reach the community of Jean Lafitte, the first thing you notice is that most buildings are elevated, either on stilts, pilings, or mounds of earth. Houses are required by local regulations to be built at least 9 ft above sea level. Bulkheads reinforce the edges of the Gulf Intracoastal Waterway and Bayou Barataria. The land is only 3 ft above sea level, and coastal communities are at constant risk from multiple climatic and environmental threats, including erosion, rising sea levels, hurricanes, flooding, saltwater intrusion, and loss of marshland to invasive species, all of which are being exacerbated by anthropogenic climate change (Figure 1).

The next thing that you notice is that the majority of the businesses are related to fishing: marinas, seafood retailers, fuel for boats. Fishing is the origin of this community, which is named after the French privateer and smuggler Jean Lafitte (1780–1823). Behind the docks, trawlers, and marinas, tankers and barges lurked on the horizon of the water. After the fishing industry, came the oil industry. The people I spoke to had parents, sometimes siblings, who had jobs on oil rigs, in shipping, in one case a call centre for the energy corporation Entergy. Oil formed a potent symbol of Louisiana’s economic force, particularly in providing jobs for young white men, that sustained support for the industry despite its costs. Oil meant power and prosperity, closely tied to a certain identity formation that Cara Daggett (2018: 29) calls ‘petro-masculinity’.

However, my interlocutors did not work oil jobs, they worked in support services, such as telecommunications, automotive repair, health supplements, or in local bars, restaurants, or schools. This shift in employment highlighted a generational switch from twenty to thirty years ago when the oil industry in coastal Louisiana was booming. Residents received money from oil companies in exchange for the mineral rights of the ground under their homes, the extraction of which has left scars across the land, disrupted barrier islands, and increased the fragility of the constantly shifting coastal landscape (Austin 2006). Today there is very little active exploration and extraction of oil along the coast. The big companies have extracted what they could and sold their remaining rights to smaller companies (Theriot 2016). Corporations such as BP, Exxon, and Shell have moved into deeper waters in the Gulf of Mexico, where ultra-deep-sea drilling remains marginally profitable, but dangerous, especially for the environment (Bond 2013). The residents of Jean Lafitte remembered well the effects of the BP oil spill from the Deepwater Horizon rig in 2010, especially on the fishing industry, as it prevented any fishing along the coast for over a year. Yet still the oil industry maintained its symbolic status. ‘People need power’, I was told, ‘we gotta keep the lights on’. This was said with a shrug, indicating a level of resignation over the loss of land and pollution from oil spills as the price to pay for the benefits that fossil fuels brought, a similar expression that Arlie Hochschild (2016) observed in Lake Charles in southwest Louisiana.

In Jean Lafitte, anthropogenic climate change and the culpability of the oil industry for the titanic increase in carbon emissions over the past few decades was not a topic of everyday conversation. When I did ask directly, there was a naturalisation of climate impacts: it’s always been hot, there have always been storms. But this is not to say that the people I spoke to were ignorant of environmental conditions. They had much to say about pollution from litter and oil spills and coastal erosion. Environmental problems were conceptualised in terms of what they could see and had experienced personally. The way they constituted their climate knowledge gave rise to what I came to call radical empiricism, the first component of the cultural epistemology I trace in this article.

Radical empiricism was evident in the interviews I conducted in Jean Lafitte. I interviewed James,^4^ who was white, in his 60s, and the owner of a telecommunications company. His house sat along an inlet to a canal with a ground level that was too expensive to insure because of the likelihood of flooding during hurricanes. It had flooded twice during the time he had lived there, during Hurricanes Rita and Isaac. On the subject of why he did not believe in climate change, he said:

I track a normal tide in our canal by our neighbour’s drainpipe across the street, and when the water’s right at his drainpipe that’s our normal high tide, and it’s never changed, the water’s no higher, it doesn’t get any higher, unless we have a storm, but they keep claiming sea levels are rising, but I don’t know where to prove it.

James did not see the proof visibly on his dock, so he rejected the association between increases in atmospheric carbon levels and sea level rise, although he conceded that he had not paid attention to what he called ‘the graphs’. Even though James held out the possibility that it was getting warmer every summer, he located the reason within his own corporeal experience, as he became more sensitive to the temperature with age.

Another interlocutor considered the climate to be the same because there was ‘same amount of rain, same amount of wind … I don’t see anything different’. Yet another dismissed ‘talk about global warming’, because where she lived it was still as hot as it ever was, ‘I don’t think here it’s much of a difference’. ‘Global warming’ as a category belonged to a different paradigm; it was known about but did not hold any meaning, it was just something other people talked about. Not seeing any difference chimed in a broad sense with James’ specific observations of the water level on his neighbours’ dock. Difference had to be seen to be believed; this is the core of what I call radical empiricism. Climate was something to be lived with, water came in with storms and seasonal flooding, and then it went out again, and that was simply something they had to deal with living on the coast. My interlocutors’ conceptualisation of climate changes accords with Shirley Fiske’s (2016: 327) observation of farmers in Maryland who epistemologically incorporated personally observed patterns and cycles of nature while rejecting the ‘canonical theory of climate change’ that it is anthropogenic and causing rapid, global changes, such as sea level rise. Global warming or climate change were concepts that belonged to a different, incommensurable paradigm.

On the subject of erosion, however, James was clear-sighted: ‘the erosion’s killing us’, he told me, reflecting on the changes he had seen in his lifetime: ‘I saw the environment change from a young age to what it is now … all the places I used to fish are no longer there, it’s just open water’. Erosion was a real impact because he had seen it happen, unlike effects attributed to anthropogenic climate change. An oft-repeated statistic I heard from the bayou residents was that Louisiana loses a football field’s worth of land from its coast every 100 min. As one interlocutor observed, ‘the Gulf will be in our backdoor soon’. There was an acute awareness of the fragility of the land they lived on and the risks to their way of life that they could observe and experience personally.

Erosion, hurricanes, and flooding were ongoing environmental hazards of which my interlocutors were well aware. The bayou community is outside of the levee protection zone that has been built up in the years after Hurricane Katrina. In 2021, just three years after my research, Hurricane Ida, a category four, overtopped the levee at Jean Lafitte and caused extensive damage (Mehta *et al*. 2021). Building higher levees continues a favoured form of development project to adapt to climate impacts, such as stronger storm surges. However, building levees, alongside oil drilling and dredging navigation canals, exacerbates erosion, which has reduced natural storm protection from coastal wetlands along the Gulf Coast (Bullard & Wright 2012: 73). In development discourses, climate change has become the preoccupying concern and reason for funding projects, encouraging stakeholders to frame projects in terms of adaptation to climate change. Dewan (2022: 540) describes climate change as a ‘spice’, something added to increase appeal to development funders, that can result in ‘climate reductive translations’. Thinking only in terms of anthropogenic climate change can draw focus from, and even exacerbate, other environmental changes from human development. Climate adaptation, such as building higher levees, can produce its own forms of vulnerability (Vaughn 2022: 10).

People in Jean Lafitte knew they were vulnerable; however, their own direct observation and personal experience was prior to, and more important than, the computational models and numerical abstractions – ‘the graphs’ – of climate science. Climate changes such as erosion and oil spills could be seen, whereas sea level rise could not. I call this radical empiricism because it prioritises direct, personal experience, yet the analysis cannot end there. Why anthropogenic climate change remains incommensurable with cultural epistemologies requires further explication beyond radical empiricism. The sources of expertise, knowledge, and authority must also be addressed.

## Normal Fires Don’t Burn that Hot: Conspiracy and Complicity in Northern Arizona

Arriving in Arizona in late July of 2018, there was an excessive heat warning in effect as the temperature threatened to reach 120°F/49°C. The plane injected a jolt of ice-cold air into the cabin as we landed, as that model of aircraft had a maximum operating temperature of 118°F. Driving up from Phoenix to Flagstaff on I-17, I passed a forest fire. Lightning had ignited the tinder dry ponderosa pines of the Coconino National Forest. Two days later the monsoon rains started, and a state of emergency was declared across Coconino County due to heavy flooding. I drove down I-40 to the Hualapai Mountain Range, just outside of Kingman, for a Republican Party picnic in Mohave County.

**Figure 2.**
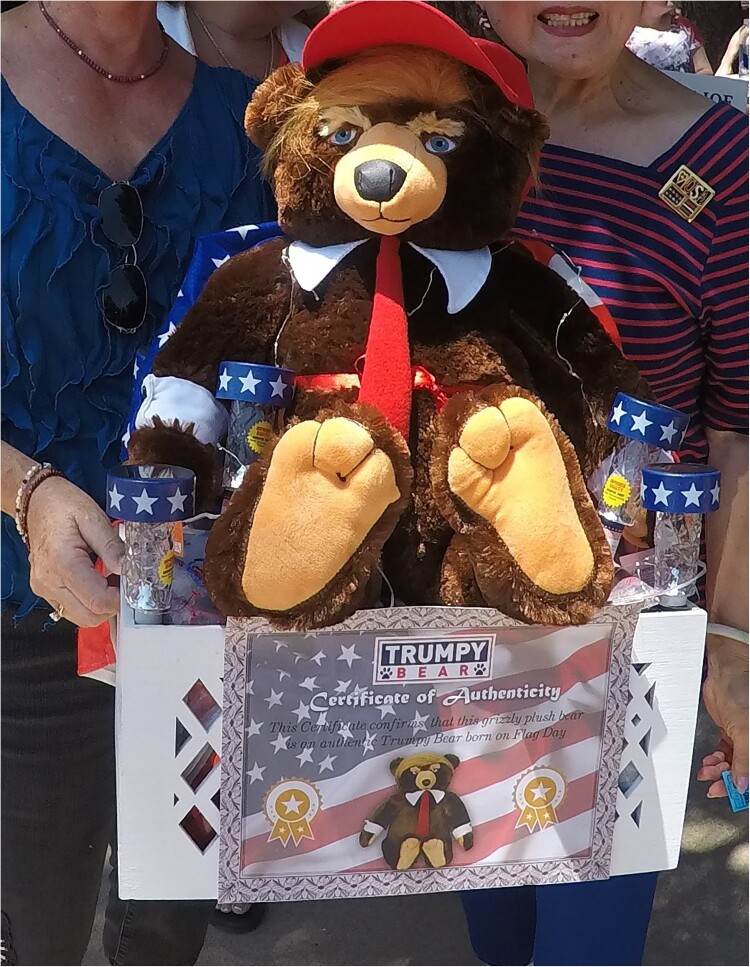
Trump merchandise at the Mojave County Republican Picnic. Author copyright.

Candidates gave speeches for the upcoming midterm elections for the open Senate seat right down the ballot to the Corporation Commission and the school board. I was there to talk to people about climate change. Ron, a man in his early 60s, author of conservative popular history books and associated with the Heartland Institute, a libertarian think tank, was sitting at a stall with his books for sale. In response to my question, he told me that he did not have an opinion on anthropogenic climate change because he did not believe in it, and he did not care about it. It was a hoax pushed by the Democrats and other ‘globalists’ to impose socialism on America. At another stall selling jewellery, hats, and t-shirts, I met Belinda, in her 70s, of the London Bridge Republican Women club. She told me that anthropogenic climate change was not happening because it was all up to God. Only he knew the day and the hour, and anyone claiming any knowledge of what would happen was trying to take God’s place, which was a sin. God would take care of us, she assured me, he gave us this world to use as we saw fit. Eric, a man in his 60s, who lived in Kingman, previously flew private planes for a company that moved musicians on tour around the country. He thought human activity was not as significant on CO_2_ levels as volcanoes (Figure 2).

These conversations presented various climate arguments that had meaning in the political-media ecosystem, the second component of the cultural epistemology underlying denial. Political – that it was just a plot cooked up by the Democrats to force through socialist policies by inventing a phoney crisis to solve. Religious – that what happened in nature could only be determined by God not affected by human activity. Scientific – that the observed changes were real but could be accounted for by other natural phenomena, such as volcanoes. They reflect a narrative that has been pushed by an entire political-media ecosystem of contrarian scientists, libertarian think tanks, cable television and YouTube channels, talk radio hosts, websites and blogs that consistently produce multiple rebuttals against climate science (Dunlap & McCright 2011; Lahsen 2015: 226–230; Bacon 2016).

Climate denial discourse is not local, according to Jonas Anshelm and Martin Hultman (2014: 112), but a transnational network driven by elite elderly men with prior success in academia or industry and libertarian think tanks. Their conclusions correlate with the explanatory framework offered by Naomi Oreskes and Erik Conway (2010), for whom climate denial is a politically constructed form of ignorance. Fossil fuel companies fund disinformation campaigns spreading false narratives about the existence or causes of anthropogenic climate change to muddy the waters of public debate. They paid scientists such as Fred Singer, an atmospheric physicist influential in the Reagan administration. Singer had previously claimed that smoking did not cause cancer while leaving his payments from tobacco companies undeclared, and then published articles and gave congressional testimony contradicting the growing consensus on anthropogenic climate change. This body of literature protected the companies from liability for the effects of their products on the planet, despite their own scientists being fully aware of anthropogenic climate change since the 1960s. People were led astray by this malfeasance, perpetrated to protect the fossil fuel companies’ profits.

What Oreskes and Conway (2010) describe is nothing less than a crime, undermining the ability of governments to respond effectively to climate change through eroding public support for the science behind it. Ron’s answer to my question about climate change was the product of this political-media ecosystem. He simply did not believe it; it was a hoax. Any observable changes have other scientific causes, such as sunspots or volcanoes. But also, the measurements of those changes cannot be trusted because they were not reliable. Ron was repeating what Fred Singer testified to Congress in the 1990s that the increase in temperature associated with global warming was simply not happening. Through emphasising the social construction of ignorance by a conspiracy of elites telling lies to dupe the masses, Oreskes and Conway ironically mirror the idea that climate change is a hoax created by a self-serving elite. In both explanations, the masses believe what they are told because they are given false information or because they are afraid.

The conservative political complexion of this narrative is clear in Oreskes and Conway’s (2010) account, in which they describe how Singer believed government intervention to help the environment was an ineffective waste, and therefore anyone proposing it must have an ulterior motive. The Heartland Institute that Ron sometimes worked for, giving talks at their office in Arlington Heights, is part of the political-media ecosystem. Heartland received funding as a registered charity from Exxon Mobil, Koch Industries, as well as Phillip Morris, the tobacco company.^5^ Eric’s response also invoked alternative explanations for observed changes in the atmosphere, blaming volcanoes.^6^ Sunspots and volcanoes persist as talking points in the political-media ecosystem which employs a range of tactics, from outright denial that global warming is happening to alternative scientific theories for why the observed changes are occurring.

When I spoke to long-term interlocutors in Arizona about climate change, they immediately interpreted the term in the frame of chemtrails (Crockford 2021: 157–164). Chemtrails were described as the emissions of aeroplanes that spread out in the sky and lasted longer than ordinary contrail emissions because they were laced with what were called ‘heavy metals’ such as aluminium, barium, and sulphur dioxide that fell to the ground from high altitudes poisoning the people breathing the air below. The government was held responsible; they were using these particles to keep us weak, sick, perhaps to even control our minds or alter the weather. Climate knowledge was constituted conspiratorially, interpreting observed changes as an attack directed by their own government.

It is instructive to juxtapose the conspiracy theory of chemtrails with the approach of Oreskes and Conway (2010). What they describe in the actions of fossil fuel companies and scientists such as Fred Singer could also be called a conspiracy. The outcome of this conspiracy was a confusion of alternate explanations that undermine alignment with mainstream science and political action on anthropogenic climate change. In this reading, there are good scientists, trying to spread the truth, versus bad scientists, paid for by the fossil fuel companies. That their explanation mirrors that of scientists denying anthropogenic climate change can inadvertently lead to a false equivalence. Unsure of how to balance competing claims, lay people regard both sides with suspicion. Chemtrail theories suggest all such elites are corrupt. The merging of anthropogenic climate change with chemtrails further blurs the coherence of the concept of ‘climate change’. In this political-media ecosystem, the impacts of anthropogenic climate change, such as devastating forest fires, are reinterpreted as evidence of high energy beam weapons, vaporising homes, cars, and entire towns. Because, they claim, no forest fire could burn that fast and that hot.

It is perhaps straightforward to interpret theories of chemtrails and high energy beam weapons as clearly false. Yet, what is happening to the Earth currently is so unprecedented that many struggle to understand and process what is going on around them. There really are devastating fires and floods occurring, yet business as usual continues, despite the dissolution of atmospheric normality as humans have experienced it for centuries. Going back to the Republican Party picnic in Mohave County, anthropogenic climate change was not spoken of by any of the candidates for elected office, but its impacts were, particularly the availability of water. Mohave County is on the edge of the Mojave Desert, it has very little freshwater, and relies mainly on groundwater reserves. The candidates complained of Californian companies stealing their water, of the big cities in Arizona, Phoenix and Tucson, taking their water. California, Tucson, and Phoenix are racially coded, ‘Latino’ or ‘Mexican’ areas for conservatives in rural Arizona. They did not connect the depletion of groundwater aquifers to the global water crisis caused by agribusiness overuse and rising temperatures (Bessire 2022: 2–3). The conflict was over *who* used the water, not whether the water should be used. The people at that picnic spoke of a bleak future where they would have to fight for water, where there was a ‘war’ against them and their way of life by politically and racially coded others. That story of being under attack by hostile forces made sense of the climate changes they witnessed, within the parameters of their cultural epistemology. The political-media ecosystem buttressed these explanations, using paid experts to produce alternative theories, but also through maintaining a specific sense of order, to which I turn next.

## There’s No Changing Nature: Opposition and Order in Central Missouri

Driving out of St Louis heading west on I-44 towards Springfield and the Ozarks, I passed a roadside chapel for truckers. This part of Missouri is heavily forested, with lush, verdant trees filling the banks of the highway for miles. Listening to AM Radio, a recording of a church service played with the speaker talking about funerals as a sombre occasion to remind the living that the dead have no more opportunities to serve Christ, only the living can do that. The living must ‘adorn their resume with gold and jewels’, the dead have already sealed their fate. Funerals are a good time to minister or witness to the living, to remind them to serve Christ while they can. Through these words, I heard intimations of what I call a cosmological schema, the third component of the cultural epistemology, that naturalises the proper order of God, humans, and nature.

Leaving I-44, through farmland dotted with baled hay, I stopped in Vienna, in Maries County. There was a small antiques fair and farmers market on a bare patch of grass, and behind it a second-hand store. Inside the store, I interviewed Evelyn, who told me she did not know what I meant by climate change. I asked instead about global warming, which she had heard of, but she thought it was ‘overblown’. Really the problem was overpopulation, in her opinion, but it was not as bad as people said. She had heard it was getting hotter, but it had always been hot. And times had been harder in the past. At 85 years old, she had lived through the Depression. Raised in a farming community, she was the child of farmers, who worked 40 acres with their 9 children. She grew up and worked in the same small town, as the postmaster and as a wife and homemaker. She felt blessed. As a child, they were very poor, but they did not know they were poor because everyone in the town was the same. Her father used to feed leaves to the cows because there was no grass. People now did not have it so hard, but they complained more.

Evelyn attended the Church of Christ in Vienna, which described itself as a Restorationist Christian congregationalist church, with an emphasis on the Bible. It took a literalist stance on biblical interpretation and could be categorised more broadly as conservative evangelical Protestant. Her answers were infused with this religious perspective, telling me that nature could not be changed, that God was in charge, and everything is ‘God’s creation’. She paraphrased the book of Genesis, saying that Adam had dominion over the animals and the Earth. Her answers closely resembled those of Belinda, in Arizona, who considered the climate as God’s responsibility. In these and other answers from all three states, I read a cosmological schema of God, who is in charge, then humans, then nature. God has ultimate agency, humans have personal responsibility, and can use nature to provide for themselves. Beyond provisioning humans, nature would be how it is and no more; it was prima facie. This position reflected what Veldman (2019: 48) described among conservative Protestants in Georgia who saw worrying about the environment on a wider scale as lacking faith in God. God was the proper cosmological position for determining solutions to global problems, not human beings.

This cosmological schema was leveraged by spokespeople in the political-media ecosystem shifting a prior commitment to environmental stewardship among Protestant evangelicals into a rejection of anthropogenic climate change that accorded with the interests of their corporate backers (Pogue 2022: 6–7). The Heartland Institute operates as a nexus point in this ecosystem, holding talks, disseminating print literature and online content, hosting fellows to do research. Noah, a research fellow, who had a PhD in environmental ethics, explained climate change to me in an interview as an invariable aspect of geological time and claimed that human contributions to recently observed fluctuations in temperature were still an ‘open question’. He took what he called a ‘God’s eye view’ of climate to undermine the idea that humans could possibly know what the ideal climate for human life was; significantly, he called this ‘pure hubris on people’s part’. Climate science was people playing God. In this way, he used themes found in conservative Christianity to buttress politically motivated denial. Humans are not God, and climate scientists try to displace God when they say they know what will happen, and how the world will end.

Noah’s description of anthropogenic climate change as ‘hubris’ is particularly revealing, connecting climate science with the sin of thinking humans can have knowledge on par with that of God. In this context, it is an offence against God to think you know things about the climate and why whatever is happening in the atmosphere is happening. This framing also reinforces radical empiricism; knowledge of the climate is not possible because it is beyond the extent of human experience. Instead, humans should have faith in God that he will take care of them. A similar conclusion is drawn by Veldman (2019: 48) who found that structural arguments concerning environmental issues were taken as an affront to God by her interlocutors in Georgia; they held faith as more important than knowledge. Religious arguments have value in this cultural epistemology, but using God in arguments makes no sense in climate science, illustrating how these different paradigms are incommensurable.

This consistent framing that I heard among people in Missouri, Arizona, and Louisiana extended beyond those like Evelyn who identified as active church members, to those who identified with a broadly cultural Christian nationalism that did not require church attendance or self-conscious religious belief (see Du Mez 2021). The cosmological schema of God-Humans-Nature in a descending hierarchical order was maintained throughout. God has ultimate power; humans have free will and dominion over nature. Humans do not always make good choices, and therefore are able to make things better or worse. However, anthropogenic climate change as a theory attributed too much power and agency to humans. Nature was how it was, self-evidently, and only God had the power to enact changes in nature, rendering human causation of rapid, global climate change incommensurable with their cultural epistemology.

In House Springs, Jefferson County, I spoke with Hank, who said that humans were too insignificant to affect the climate on any scale, it was all up to ‘Mother Nature’. His father had lost two farms in Iowa during the Depression and had spent the rest of his life as a sharecropper. Hank spent his career driving trucks, long and short distances. Raised Methodist, Hank stopped going to church after he married. He expressed sentiments in line with the cosmological schema that Evelyn’s responses also indicated, that ‘Mother Nature’ determined whether it was hotter or colder, and that humans had no ability to alter or affect the climate. Rather than climate nihilism, Hank’s understanding expressed a similar cultural epistemology as my Louisiana interlocutors and Fiske’s (2016) descriptions of Maryland farmers, in which the patterns of nature were closely observed but ultimate had to be lived with, as altering those patterns was beyond human capacity. Living with the climate, rather than being in control of it, was the role of humans in this cosmological schema.

Hank’s response that ‘Mother Nature’ was responsible for the climate also echoed closely the points in a policy document that Fred Singer published with the Heartland Institute: *Nature, Not Human Activity, Rules the Climate* (2008)*.* Like Evelyn, Hank also reminisced about the past, when people did not complain so much and worked harder, and also that farming was a ‘hard life’ in which ‘you’re at Mother Nature’s mercy’. This sense of human insignificance coupled with the primacy of personal responsibility evokes the cosmological schema described above. God is ultimately in control, at the top, with humans underneath, responsible only for their own actions, and then nature as separated from humans and largely self-regulating. The effect of this schema was to render action to improve the environment limited to what humans needed to provision themselves. The question then remained as to why anyone would support action that would only be a waste of time and resources. There had to be another explanation. It was this suspicion that the political-media ecosystem leveraged, shifting from asking questions to forming conspiracy theories.

Hank in many ways fits the characterisation of a ‘cool dude’ in McCright and Dunlap’s (2011) work, in which they found that conservative white men in the US were significantly more likely to deny anthropogenic climate change. White conservative men perceived less risk because they were on average less vulnerable to risk, cohering with Sarah Vaughn’s (2022) argument that race mediates vulnerability to climate impacts. Similar to my Louisiana interlocutors’ support for oil, Hank’s argument for continuing use of coal was that many people’s livelihoods were dependent on coal. Discontinuing coal would undermine economic prosperity. Anthropogenic climate change undermines and even sabotages the legitimacy of the prosperity that older American conservative white men such as Hank benefitted from; it also threatens their identity. Fossil fuels form part of what Cara Daggett (2018: 29) names petro-masculinity, in which conservative male identity interlinks with authoritarian politics. Anthropogenic climate change implies American capitalist prosperity came at an unimaginable price: the destruction of the future. So, older white conservative men justify and defend the status quo that benefits them. In the political-media ecosystem, they see other white male conservatives in their in-group doing the same, such as talk radio hosts, Fox News anchors, and Republican politicians (McCright & Dunlap 2011: 1165). These spokespeople reinforce a cultural epistemology that is incommensurable with anthropogenic climate change.

Anthropogenic climate change involves transformative impacts on global, atmospheric, oceanic scales but not in Hank’s house. Yet there is talk of this immense, catastrophic change, and people say the American way of living is responsible. By refusing to accept it, Hank and others like him can claim a sense of innocence. The connection raised in the introduction between climate denial and denial of systemic racism is not incidental or analogical but historically contingent, part of the same processes (Yusoff 2019). The colonial practices of extraction, of which slavery was a tool, and anthropogenic climate change an effect, required wilful silence about their existence, of how bodies and rocks were made inhuman matter to power prosperity for a select few (Hartman 2007: 15; Yusoff 2019: 16). What Norgaard calls the ‘the social construction of innocence’ (2011: 213) underlying climate denial is but a vein of the interconnected circulatory system of white innocence through which capitalist colonial extraction runs. Gloria Wekker (2016) unflinchingly names how a denial of racism is central to a form of ‘self’ that imperialism produces. Whiteness is not acknowledged as a racial positioning because of the long history of imperialism and the role it played in processes of meaning making, including self-formation, rendering whiteness as an invisibilised norm (Wekker 2016: 23). Colonialism and racial capitalism are co-constitutive and require denial to continue to operate.

Denial requires and allows white people to be unaware of their whiteness, while benefiting immensely from its political and economic privileges. Privilege maintenance composes part of the ‘armor’ of climate denial identified by Norgaard (2011: 217). Systems of supremacy are invisible to those whom they privilege; denial is a necessary camouflage that allows supremacism to operate and perpetrators to position themselves as victims (Mills 1997: 2). Denial produces a militant ignorance that seeks to destroy the knowledge that would reveal its hollow core. Acknowledging climate change would mean acknowledging the extraction and genocide that lead to American prosperity.

In 2019, the following spring after my research, the Missouri River flooded. Across the Midwest, including Central Missouri, the period of January to May of that year was the wettest on record, with the amount of rainfall likely exacerbated by anthropogenic climate change (Almukhtar *et al*. 2019). Major floods occurred throughout the Missouri and Mississippi river system, and delayed or disrupted entirely the planting season, which farmers recorded on social media under the hashtag #NoPlant19 (Houck 2019). The consequence of a disrupted planting season is an increase in scarcity in the food supply. Anthropogenic climate change has compounding effects that emerge through complex systems, in which causality is not easily assessed. Extreme events like the flooding of 2019 are absorbed into a cultural epistemology of constant climatic variability beyond human control. What people could control was how they dealt with these problems, just as they had in the past. People who had survived the Depression through what they understood as their own individual effort and the grace of God continued to live by those stories that emphasise personal responsibility and faith in divinity over scientific theories and collective solutions to structural problems. The stories told of the past reflect projections of the future. In their cultural epistemology, there is no conceptual space for a dying world that they are complicit in killing; it remains an incommensurable proposition.

## Conclusion: A Cumulation of Crises

From 2018 to 2022, opinion polls in the US indicate that belief in climate change has risen from around 50% to around 70% (Leiserowitz *et al*. 2023: 3). During that same period, carbon emissions have continued to increase precipitously. Belief, knowledge, and action do not necessarily connect in legible ways. The unstable boundaries of knowledge continually shift, yet what remains is a tendency to normalise a reality in which everything is fine (Norgaard 2011: 207). My argument is that this normalisation comes not from emotional reactions or information deficit but because cultural epistemologies are insufficient for the scale of the problem faced. Anthropogenic climate change is an excessive problem, an excess of carbon dioxide, an excess of extraction and production; it exists at scales that exceed cultural epistemologies.

A common accusation against conservatives in the US South is that they do not care about the environment. However, my ethnographic engagement indicates that they do care. They care about erosion and storms and fire and flood; they care about their homes and their livelihoods and their communities. They are aware of climate changes and impacts from extreme weather events. However, they understand these events in a different cultural epistemology of nature and climate than the consensus framework of climate science, one that prioritises natural and supernatural causation for climate changes they experience. This is how a white man in Louisiana can tell me with complete sincerity that he is both a strong environmentalist and a big supporter of the oil and gas industry. Like all cultural subjects, my interlocutors lacked coherence between knowledge, belief, and actions (Lennon 2018: 442). My interlocutors held the damage to their environments and their support for fossil fuels in tandem. Understanding does not necessarily lead to caring, and caring does not necessarily lead to action. Norgaard’s (2011) Norwegian interlocutors cared and understood, but their action was on par with the Americans I spoke to. There is an assumption in political activism that individual awareness will lead to social change, based on the idea that individuals have the power to change society. This assumption leads to a focus on consumption over production, on voting over regulation.

Radical empiricism prioritises knowledge gleaned from personal experience. A neoliberal primacy of the individual, lifting one’s own opinion and experience out of collective positionality, is buttressed through a cosmological schema that situates humans between God and nature, dependent on one and independent from the other. This schema is leveraged by a political-media ecosystem that is cannily aware of how its audience thinks and speaks in the language that they will understand and respond to. This ecosystem exacerbates the isolation of the individual and their experience and their opinion, with any conflict blanked out as ‘just politics’ or people ‘complaining too much’. A trick of this ecosystem is how it has made its political operations seem apolitical, instead labelled as a form of ‘culture war’. This drives a sense of antagonism, that there are two sides implacably opposed, unable to find common ground because the threat posed by the other side is perceived as existential. Polarisation as a narrative flattens nuance and accentuates difference.

The ongoing catastrophe that is climate change reveals deep rifts between cultural epistemologies and climate science, paradigms that exist in the same social reality but that are incommensurable. The ensuing epistemological crisis will likely produce a profound paradigm shift. The good news is that as the climate changes, people are changing their minds. To help this process, a useful approach is to frame climate solutions in terms that make sense to local peoples. In the US South, this can mean emphasising energy independence and environmental stewardship. However, because of the complex epistemological dynamics outlined in this article, the political-media ecosystem also needs to shift to support climate action, and their corporate backers held accountable for the damage they have wrought. But that would require addressing the root causes of climate change and American prosperity and disavowing both.

## Notes

1 This article does not have the space to expand on the links between covid-19, vaccines, and climate change as various fronts in an interlinked crisis of trust and authority in institutions and experts, however, see Crockford (2022).

2 https://www.carbonbrief.org/analysis-which-countries-are-historically-responsible-for-climate-change.

3 Interviews pertaining to climate change were undertaken in 2018 across all three states, in rural areas, with majority white populations, previously identified using Yale Program on Climate Communication data on levels of climate denial. While I did not intend to interview only people who identified as Christian, those who rejected the idea of climate change also identified as practising Christians or as being raised in the church.

4 All personal names are pseudonyms.

5 It has received at least $676,500 since 1998 from Exxon https://www.desmog.com/heartland-institute/.

6 While volcanoes do release carbon dioxide, human activity contributes 100 times the impact of volcanoes to the carbon cycle, according to NASA: https://climate.nasa.gov/faq/42/what-do-volcanoes-have-to-do-with-climate-change/.
